# Impact of debt, reserves, and political stability on Sri Lanka’s financial crisis

**DOI:** 10.1371/journal.pone.0294455

**Published:** 2023-11-17

**Authors:** Candauda Arachchige Saliya

**Affiliations:** Department of Business Management, SLIIT Business School, Sri Lanka Institute of Information Technology, Colombo, Sri Lanka; Universiti Malaysia Sabah, MALAYSIA

## Abstract

This study attempts to explore the impact of external debt ($Debt), foreign reserves ($Reserves), and political stability & absence of violence/terrorism (PS&AVT) on the current financial crisis in Sri Lanka. Using data from 1996 to 2022 obtained from the World Bank (WB) and the Central Bank of Sri Lanka (CBSL), a regression analysis is conducted, with a composite variable named "CRISIS," which accounts for interest rate, inflation, currency devaluation adjusted to GDP growth, as the dependent variable. The findings indicate that, collectively, these predictors significantly contribute to explaining the variance in the financial crisis, although their impact is relatively minor. While the direct influence of PS&AVT on the financial crisis is not statistically significant, it indirectly affects the crisis through its considerable impact on debt and reserves. Granger causality tests showed predictive value for $Debt and $Reserve in relation to CRISIS, but the reverse relationship was not significant. Regression analysis using the error term and scatter plots supports the absence of endogeneity issues in the model. These findings suggest that while external debt and foreign reserves are more directly related to financial crises, political stability and the absence of violence/terrorism can influence the crisis indirectly through their effects on debt accumulation and reserve levels. This study represents a pioneering effort in investigating the impact of external debt, foreign reserves, and political stability on the financial crises in Sri Lanka. By utilizing a comprehensive dataset and applying a regression analysis, it sheds light on the complex interactions between these variables and their influence on the country’s financial stability.

## Introduction

In the field of economics, understanding the intricate relationships between key macroeconomic variables such as inflation, interest rates, exchange rates, debt, foreign reserves and economic growth is essential for effective policy formulation and decision-making. The Covid-19 pandemic has sparked renewed interest in sovereign debt crises, as demands for debt relief for developing nations have emerged. Therefore, it is crucial to contextualize recent research findings in this field within the broader economic landscape, particularly in light of the growing concerns surrounding sovereign debt crises in various nations, including Sri Lanka. A report from the International Debt Statistics (IDS) 2021 reveals that prior to the pandemic, many countries already had high levels of debt to the tune of $8.1 trillion. Many of these countries, including Sri Lanka, have debt to GDP ratios surpassed 100%. These Figures underscore the ominous reality that debt burdens had become increasingly unsustainable, despite two decades of debt relief initiatives led by the World Bank in collaboration with the IMF and the international community [1].

Sri Lanka is currently experiencing a grave political and financial crisis, which has resulted in millions of people being unable to afford basic necessities such as food and medicine [2, 3]. The nationwide protests against the escalating cost-of-living crisis climaxed with the storming of the presidential palace on 9^th^ July 2022 and the resignation of both the President and his brother Prime Minister. To mitigate the crisis, the Sri Lankan government has again sought assistance from the International Monetary Fund (IMF). In May 2022, the island nation defaulted on US$51 billion of external debt ($Debt) for the first time in Sri Lankan history. In 2022 alone, Sri Lanka faced debt obligations amounting to US$6 billion, while its foreign reserves ($Reserves) were only US$ 1.9 billion [4]. It is important to note that US$1.5 billion of this amount is tied up in a swap arrangement with China, which cannot be utilized for dollar payments [5]. Sri Lanka’s foreign debt depresses income and stimulates price level and have raised interest rates [6].

Further, the situation has reached such a critical state that experts predict a rise in acute malnutrition from 13 percent to 20 percent, while the number of severely malnourished children is expected to double from 35,000 to 70,000 [7]. Import restrictions on fertilizer and agrochemicals resulted in a reduction not only in domestic essential crops but also in export earnings on products such as tea, which accounts for 11% of national export income. This was further aggravated by a pegged exchange rate to the US dollar, which discouraged all sorts of exporters and inward remittances while drained off $Reserves.

With the government’s stark admission of Sri Lanka’s financial bankruptcy, the country found itself engaging in negotiations from the standpoint of a financially beleaguered nation. The President candidly acknowledged that this journey would be hard and strenuous [8].

Henceforth, a potential intricate interplay may have emerged similar to the global financial crisis of 2008, affecting numerous economies, including Sri Lanka. The escalation of government debt levels has multifaceted consequences, impacting both government deficits and shaping investor sentiment. On one side, the upsurge in government deficits heightens the necessity for borrowing, subsequently exerting upward pressure on interest rates. This, in turn, can trigger a "crowding-out" phenomenon, dampening private investment and potentially impeding overall economic growth [9]. Conversely, the heightened risk aversion among investors may favor bonds issued by countries perceived to have a lower default risk. Simultaneously, as economic growth dwindles and government revenues decline, governments face the imperative to increase their debt levels to sustain welfare programs. The convergence of these economic challenges in Sri Lanka and the pioneering research on inflation, interest rates, and economic growth in elsewhere forms a backdrop against which I explore the multifaceted dynamics of the situation and the vital role of policymakers in navigating these complex waters [10].

## Literature review

After analyzing and evaluating the current and future debt levels of 68 countries that hosted the China-funded projects, the Center for Global Development (CGD) has identified ten countries (Sri Lanka, Kyrgyzstan, Djibouti, the Maldives, Laos, Mongolia, Pakistan, Montenegro, Angola, and Tajikistan) as ‘at risk of debt distress’ [11]. However, against many predictions by industry experts, after imposing very painful conditions, IMF granted US$2.9 billion in September 2022 and approved further US$3 billion in March 2023, both under the terms of 48-months as per the Extended Fund Facility (EFF) arrangements [12, 13] (IMF 2022; 2023). The Sri Lankan Rupee depreciated against US$ by approximately 555% on an annualized basis, dropping from LKR 200.92 in February to LKR 294.00 in March 2022 and continued depreciating until hitting its lowest point at LKR 368.50 in November 2022. However, since then, The Sri Lankan Rupee has appreciated and recorded as LKR 305.00 on 24^th^ May 2023, which is an annualized appreciation of 33.7% [4, 14].

Further studies of similar experiences faced by the countries elsewhere revealed that, to arrest such crises, they had established peace immediately [15–19] and then implemented structural reforms towards liberalisation of markets [20–25]. The success of all other short-term solutions such as debt restructuring, selling state assets and controlling imports etc. seem inevitably to depend on a stable socio-political climate and policies conducive for foreign direct investments.

A long-standing stern debate prevails between *left* and *right* camps in the political arena as well as in the academia on structural reforms towards liberalization conditioned by the IMF. The main arguments of “the left camp are that liberal policies could stimulate corruption and discrimination, obstruct inclusion, empower the elite-class to accumulate wealth disproportionately, increase inequalities and also could bring adverse effects to certain traditions and sociocultural values of people” [26–31]. In contrast, “right wing argues that liberal policies stimulate economic development by removing certain traditional barriers, enable nations to create more wealth which will trickle down to everyone else, hence improve living standards in general by translating individual greed into collective good and also it would ensure law and order towards justice and diversity” [32, 33] (Bourguignon 2018; UN 2010).

Bhowmick [34] points out that the nature of borrowing and $Debt obligations are the key contributors to Sri Lanka’s current economic crisis. Good governance can be defined as the process by which governments make decisions, implement policies, and manage public resources [35]. High-cost foreign debt could trigger crises when the WGI in a country are weak [28] and there is widespread agreement that the country’s high levels of $Debt are a major contributing factor [36]. This has been aggravated by external factors such as the COVID-19 pandemic and Russia-Ukraine war which have hit the country’s tourism industry hard, as well as internal factors such as corruption and mismanagement of public funds [12].

### External debt ($Debt) and foreign reserves ($Reserves)

Although $Debt can contribute to increase $Reserves, it can cause serious difficulties, especially during crisis periods. It is widely accepted that the current financial crisis in Sri Lanka is attributed to the burden of international debt stimulated by deteriorating of $Reserves [5, 34]. While some argue that international loans, including those from the IMF, can be beneficial [8, 37], others contend that current IMF facilities may cause stress as they lack longer tenures, are not interest-free, lead to social-unrest, undermine sovereignty, perpetuate poverty and inequality, create dependency and present moral hazard among policymakers [5, 38–43].

**Fig 1:**
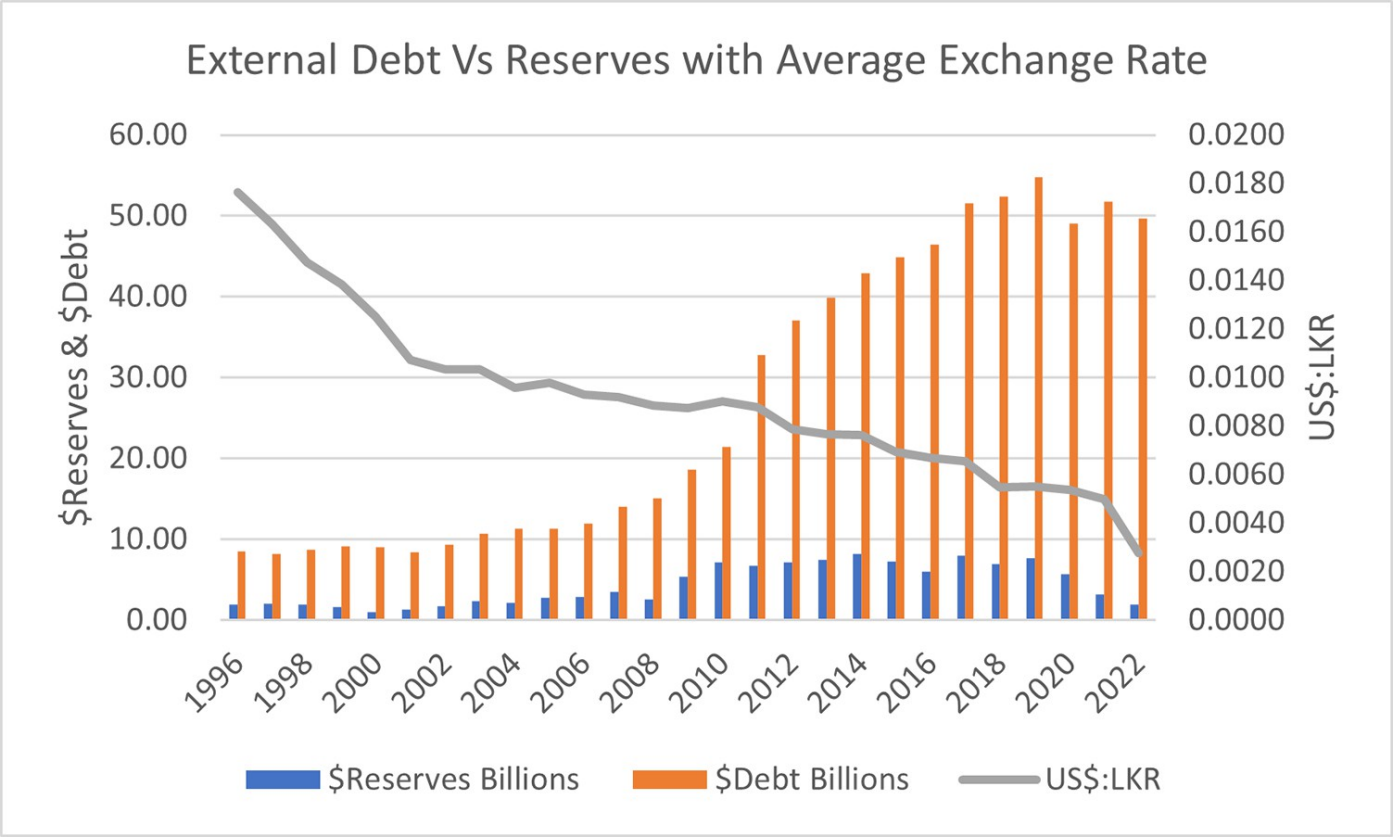
$Debt and $Reserves of Sri Lanka from 1997 to 2022 compared to the exchange rate. Sources: Author’s illustration based on data from The CBSL, The World Bank and Macrotrends.

The Fig 1 shows the accumulation of $Debt compared to $Reserves in Sri Lanka from 1997 to 2022, highlighting the concurrent weakening trend of Sri Lankan Rupee. As at end 2022, Sri Lanka’s total public debt was US$ equivalent 83.6 billion (including arrears) comprised of US$ 45.5 billion of foreign currency stock and US$ 38 billion of local currency stock of Central Government debt, including US$ 3.7 billion of guaranteed state-owned enterprises (SOEs) loans and US$ 3.1 billion of CBSL debt [4, 14].

Sri Lanka’s $Debt was 69.7% of its Gross Domestic Product (GDP: LKR 23,700 billion or US$ 65.38 billion) and its total debt amounted to a staggering 128% of its GDP [4, 12, 44]. This high level of debt has led Sri Lanka to focus its economic strategies on sectors that bring in foreign currency, primarily export industries. These industries include the production of clothes for US and European markets (which account for 52% of all Sri Lankan exports), tea, coffee, and spices for export, and tourism development [14].

The available $Reserves dropped to an unprecedented $20 million in April 2022, though official $Reserves were reported at around US$1.6 billion. This Figure includes a restrictive amount of US$1.4 billion with the People’s Bank of China that cannot be utilized to finance imports until Sri Lanka accumulates $Reserves equivalent to three months’ worth of imports (US$5.1 billion) [45]. Higher $Reserves reduce the perceived country risk, while higher $Debt increases it. However, there are also studies (for example [46]) that claim that $Reserves have a negative impact on $Debt, suggesting that countries with higher $Reserves tend to have lower levels of $Debt irrespective of their GDP per capita standing; high-income country (such as oil-exporting countries) or low-income country.

### Reflections of economic crisis

A rich body of research and academic literature spanning a significant historical timeline extensively examines the multifaceted factors that either contribute to or emerge as consequences of financial and economic crises across diverse economies. These factors encompass, among others, interest rates, inflation, exchange rate depreciation, economic contractions marked by negative GDP growth, various forms of deficits such as budget, trade, and balance of payments deficits, monetary expansion (money printing), government debt accumulation, and foreign reserve management (see [7, 20, 26, 46–48]). This comprehensive body of research provides crucial insights into the dynamics of financial and economic crises, enhancing our understanding of their causes and implications.

#### Debt and economic growth

Theoretically, both neoclassical and endogenous growth models [49, 50] suggest that high levels of public debt will always reduce the rate of economic growth. Additional channels in support of a negative effect of public debt on long-term growth include: (i) the “debt overhang” hypothesis [51] (ii) the “constraint” hypothesis (iii) the ‘crowding out’ effect.

The assertion that high levels of public debt impede GDP growth (a negative components of the composite DV-CRISIS) finds support in a growing body of empirical evidence, which illustrates a non-linear, negative relationship between public debt and economic growth in both advanced and emerging market economies [49, 50, 52–55], Gomez-Puig and Sosvilla-Rivero [56] provide evidence of a "diabolic loop" between low economic growth and high public debt levels and public debt has a detrimental impact on economic growth, particularly beyond specific debt thresholds unique to country. Findings of Panizza et al., [57] (2014) also aligns with this growing empirical literature suggesting a negative non-linear correlation between public debt and economic growth.

Some studies (for example, [58, 59]), unveil a bilateral causal relationship between debt and growth appears to be relatively weak. However, there is limited evidence of a robust, long-term causal effect when employing bivariate Granger causality tests [58]. According to Baum et al. [59], the short-run impact of debt on GDP growth is significantly positive but diminishes and loses significance beyond specific debt-to-GDP ratios, which vary between countries. Notably, when debt-to-GDP ratios exceed certain thresholds (e.g., above 95%), additional debt exerts a negative impact on economic activity.

Therefore, causal effects in either direction between debt and growth cannot be rejected across the board, it highlights the necessity of considering country-specific dynamics and nonlinear properties when conducting such analyses [58, 59].

#### Interest rate, inflation and exchange rate

Swift accumulation of debt, particularly when it culminates in a debt crisis, can potentially trigger a currency crisis. After a sovereign default, creditors may decline to extend loans and withdraw their investments due to concerns about a recession, exerting downward pressure on the exchange rate [60]. Additionally, Marques et al. [61] suggest that although debt surprises raise long-term inflation expectations in emerging market economies in a persistent way, but not in advanced economies. However, as noted by Podkaminer [62], the prevailing belief that increasing public debt inevitably results in future inflation lacks empirical support. Meanwhile, Romero and Marin [63] have found that find that for countries whose public debt is already high, further increases in public debt are inflationary.

#### Political stability

Studies have shown that political stability has a significant impact on a country’s debt accumulation [26, 64]. They argued that political instability can increase borrowing costs and reduce investor confidence, ultimately leading to higher debt. On the other hand, some other studies [26, 28] found that political stability is negatively related to debt. They argued that political stability can improve macroeconomic stability and reduce borrowing costs, leading to lower debt. However, Mehmood et al [65] argue that the impact of political stability on debt may be mediated by the level of institutional quality. They found that political stability has a positive impact on debt in countries with low institutional quality, but a negative impact on debt in countries with high institutional quality. Several studies have investigated the impact of political stability on reserves. For instance, a study by Mofady and Malawi [66] examined the impact of political instability on reserves in Jordan and found that political instability has a significant positive impact on reserves, suggesting that countries with higher political stability are likely to have higher foreign reserve levels.

Previous studies have shown that governance and political stability positively affects debt and reserves, indicating that better governance can contribute to higher levels of debt and reserves [34]. On the other hand, certain studies have shown that good governance, particularly in areas such as political stability, control of corruption, and regulatory quality, can significantly influence a country’s debt and reserves [26, 28].

All of these factors are intricately intertwined, collectively contributing to the onset of a financial crisis, which, in turn, often triggers an economic crisis marked by a contraction in the economy."

### Theoretical framework

This study builds on previous research by using updated data to examine the relationship between $Debt, $Reserves and Political Stability and Absence of Violence/Terrorism (PS&AVT) focusing on Sri Lanka. The PS&AVT is one of the World Governance Indicators (WGIs) which have been used in numerous studies in finance and economics field [67–70]. These WGIs measure perceptions of the likelihood of existence of them by an aggregate indicator, in units of a standard normal distribution. Estimate gives the country’s score on the aggregate indicator, in units of a standard normal distribution, i.e., ranging from approximately -2.5 to 2.5 [71]. For example, PS&AVT measures perceptions of the likelihood of political instability and/or politically motivated violence, including terrorism. These aggregate indicators combine the views of a large number of enterprise, citizen and expert survey respondents in industrial and developing countries. They are based on over 30 individual data sources produced by a variety of survey institutes, think tanks, non-governmental organizations, international organizations, and private sector firms [35].

#### CRISIS: The dependent variable

An economic crisis is typically characterized by several key indicators, including high interest rates and inflation, currency devaluation or exchange rate instability, elevated unemployment rates, and negative GDP growth. These factors often coincide and contribute to a challenging economic environment [72]. The dependent variable ’CRISIS’ (financial crisis) is constructed using a proxy that combines four key indicators of a country’s financial and economic situation: interest rate, inflation, currency devaluation and GDP growth because (unemployment level excluded in this study as it is more focused on a financial crisis):

- High interest rates can be indicative of tight monetary policy, which may lead to reduced borrowing and investment, potentially impacting economic growth. Additionally, high interest rates can increase borrowing costs for individuals and businesses, leading to financial stress and potential economic downturns.
- High inflation erodes the purchasing power of individuals and reduces consumer confidence. It can lead to higher production costs, decreased investment, and economic instability. Rapid or sustained increases in the inflation rate can be a sign of underlying economic imbalances and potential crisis.
- Exchange rate depreciation: The value of a country’s currency may decline rapidly during an economic crisis, leading to a crash or instability in the foreign exchange market. This can make imports more expensive, impacting businesses and consumers, and may affect the balance of trade.
- GDP growth rate: A negative or low GDP growth rate suggests a slowdown or contraction in economic activity. It may indicate declining business investment, decreased consumer spending, or reduced export demand. A sustained period of low or negative GDP growth can signal an economic crisis, as it reflects a significant downturn in overall economic performance.

By combining these factors, the equation "CRISIS = Interest rate + Inflation rate+ annual currency devaluation—GDP growth rate" provides a simple way to capture the interaction and cumulative impact of interest rates, inflation, currency devaluation and GDP growth on the overall financial crisis. Higher the value, severe the crisis. It considers both monetary and real economic factors that can contribute to financial instability and helps provide a comprehensive measure of the crisis situation.

**Table:** 

CRISIS = Interest rate + Inflation + currency devaluation - GDP growth

#### CRISIS index Vs specialized indices

**Fig 2:**
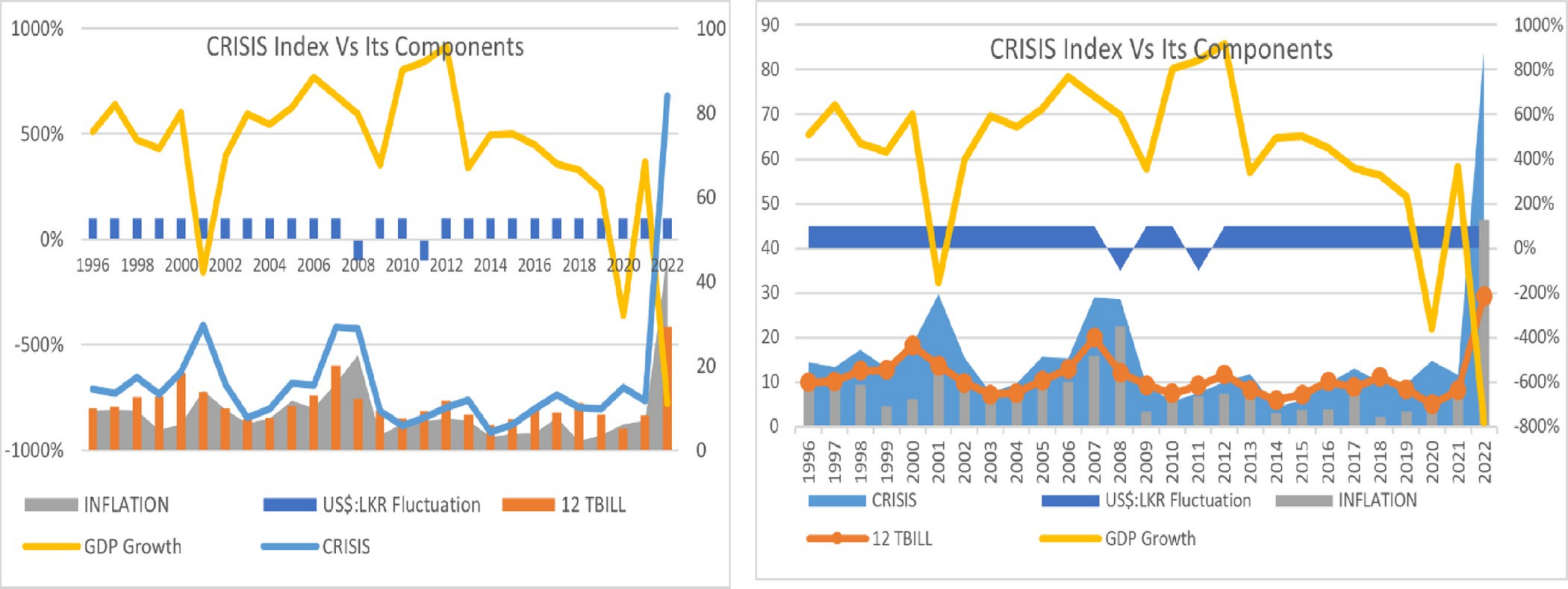
CRISIS index fairly represent the situation in Sri Lanka. Source: Author’s illustration based on data from the CBSL and the World Bank.

This CRISIS index is not meant to substitute specialized indices, such as the Financial Stress Index (FSI), Sovereign Risk Index, and Economic Vulnerability Index (EVI) which offer a more comprehensive assessment of risk and vulnerability by considering a broader range of factors beyond economic variables. They utilize rigorous methodologies, undergo validation, and provide targeted analysis of specific risks. In contrast, this CRISIS index is characterized by its simplicity, understandability, and easy applicability. It provides a quick snapshot of economic conditions, has broad applicability, and can potentially act as an early warning signal. The graphical presentation in Fig 2 shows the reflection of the CRISIS index on its components and demonstrates a fair representation of a crisis situation.

### Hypotheses

Hence, the following research hypotheses are constructed:

H1: External debt ($Debt) positively influences the CRISIS in Sri Lanka.
H2: Foreign reserves ($Reserves) negatively influence the CRISIS in Sri Lanka.
H3: PS&AVT negatively influences the CRISIS in Sri Lanka.

To examine the indirect effects of PS&AVT on the CRISIS through $Debt and $Reserves, the following hypotheses are tested:

H4: PS&AVT is positively associated with $Debt.
H5: PS&AVT is positively associated with $Reserves.

These hypotheses examine the impact of external debt ($Debt) and foreign reserves ($Reserves) on the CRISIS, as well as the role of Political Stability and Absence of Violence/Terrorism (PS&AVT) in the form of indirect effects considering the associations between PS&AVT and $Debt, as well as PS&AVT and $Reserves. This comprehensive approach seeks to provide a deeper understanding of the causes and dynamics of the economic crisis in Sri Lanka.

Afterwards, I explore the potential causes of these positive or negative influences by critically analyzing various reports, expert opinions, and scholarly works. This investigation aims to identify specific policy decisions or actions that have played a pivotal role in triggering the economic crisis in Sri Lanka.

**Fig 3:**
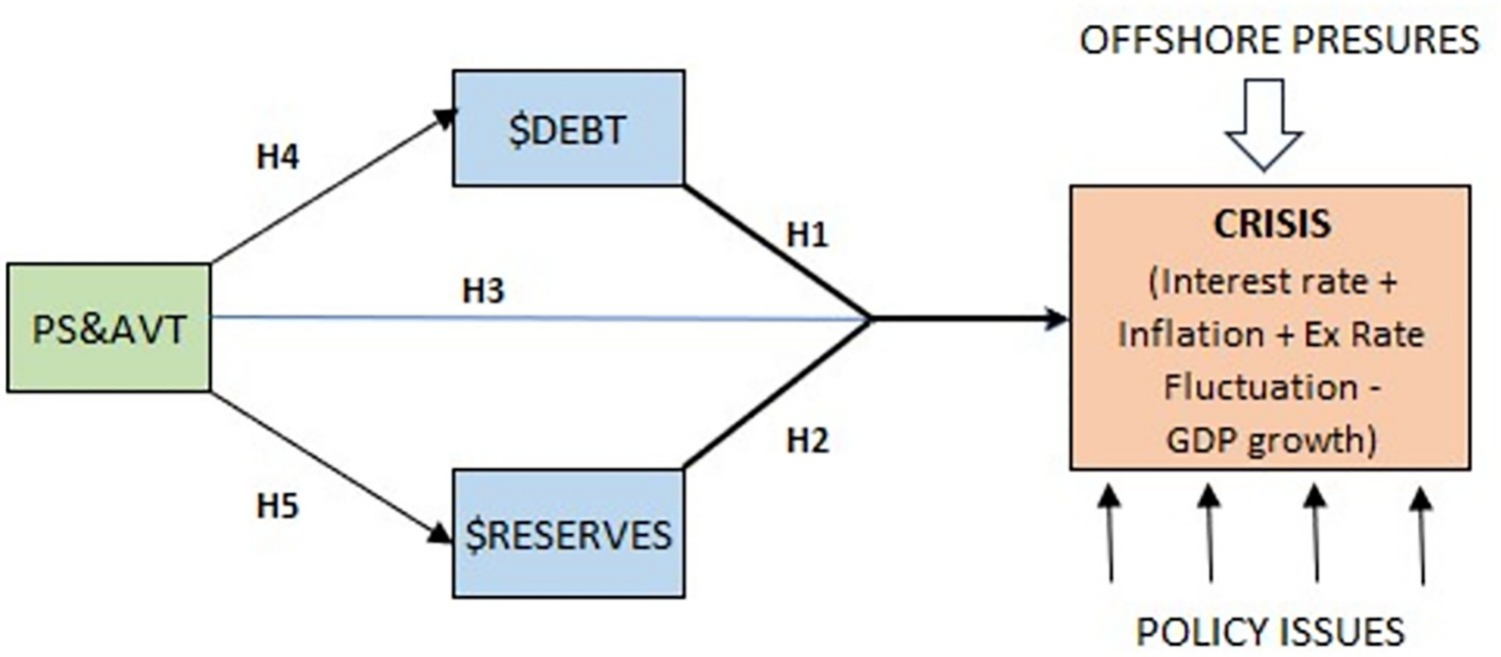
Theoretical framework.

This wholistic approach can be illustrated in Fig 3.

## Materials and methods

The method employed for data collection and analysis in this study is a flexible-integrated mixed method [27] approach that utilizes secondary data from 1996 to 2022 obtained from the World Bank Database and the Central Bank of Sri Lanka, and other authentic reports. To capture the lag effect of the independent variables on the CRISIS, the regressions were conducted with one year lag, so that, for example, the PS&AVT score, $Debt and $Reserves of 2021 are regressed on the Crisis of 2022. Therefore, for example, H1, H2 and H3 regression equation would be as follows.


$$CRISIS_{t}=\alpha +\beta_{1}\$Debt_{\left(t‐1\right)}+\beta_{2}\$Reserves_{\left(t‐1\right)}+\beta_{4}PS\&AVT_{\left(t‐1)\right)}+e$$

The regression was conducted using SPSS. Afterwards, the findings of the quantitative tests were critically analyzed with the reports of institutions such as IMF, UN entities, Reuters, Policy Institutions, The World Bank and the Central Bank of Sri Lanka (CBSL) and publicly available opinions expressed by industry experts and scholars in the field.

The dependent variable CRISIS in this analysis is constructed by combining the risk-free interest rate (1-year T-Bill), inflation (annual change of the Colombo Consumer Price Index-CCPI), annual devaluation of Sri Lankan Rupee (LKR) against US$ and GDP growth rate. In 2022, the value of the CRISIS variable is computed as 83.99 which is the highest observed in the 26-year period since 1996. According to the graph in Fig 2, this CRISIS index fairly reflects crisis situations in Sri Lanka.

Various statistical techniques were employed to assess bidirectional causality between the independent variable and the dependent variable. These techniques encompassed Granger causality tests and regressing lagged dependent variable on independent variables which provided insights into causal relationships, and regressing with the residuals (error term), combined with scatter-plots, to address potential endogeneity concerns.

## Results

### Basic characteristics

The variable CRISIS has a mean of approximately 16.17 and a standard deviation of approximately 15.30. The positive skewness (3.682) indicates a right-skewed distribution with potentially extremely high values as expected in crisis times. The high kurtosis (15.787) suggests a sharper peak and heavier tails compared to a normal distribution. All other variables demonstrate a relatively normal distribution, with skewness values ranging from 0.004 to 0.529 and kurtosis values ranging from -1.413 to -1.774 (Table 1).

**Table 1:** Descriptive statistics.

	N	Mean	Std. Deviation	Skewness		Kurtosis	
	Statistic	Statistic	Statistic	Statistic	Std. Error	Statistic	Std. Error
CRISIS	26	16.17020890	15.29934200	3.682	.456	15.787	.887
$DEBT	26	2621017987	18126981891	.529	.456	-1.479	.887
$RESERVES	26	4399243873	2538125581	.220	.456	-1.774	.887
Political Stability and Absence of Violence96	26	-.942114838	.6713651869	.004	.456	-1.413	.887

The correlation coefficient between $Debt and $Reserves is 0.784, and the correlation coefficient between $Debt and PS&AVT is 0.880, both of which are statistically significant at the 0.01 level. These findings suggest that there are significant relationships between the variables, with $Debt and PS&AVT showing stronger associations with CRISIS compared to $Reserves. However, the correlation between CRISS and PS&AVT is negative but very minimal (-.054) and not significant (Table 2).

**Table 2:** Correlations.

	CRISIS	$Debt	$Reserves	PS&AV
CRISIS	1			
$Debt	.078	1		
$Reserves	-.351	.784**	1	
PS&AV	-.054	.880**	.745**	1

**. Correlation is significant at the 0.01 level (2-tailed).

### Regressions

The ANOVA results demonstrate that the regression model, as a whole, is statistically significant. The F-statistic of 4.825 (p< 0.010) suggests that the predictor variables (PS&AVT, $Reserves, $Debt) collectively make a significant contribution to explaining the variance in the dependent variable (CRISIS). The Durbin-Watson statistic has a value of 1.716, indicating a lack of significant autocorrelation in the residuals of the regression model (Table 3).

**Table 3:** Regression.

Model		Unstandardized Coefficients		Standardized Coefficients	t	Sig.	Collinearity Statistics	
		B	Std. Error	Beta			Tolerance	VIF
1	(Constant)	11.739	14.884		.789	.439		
	$DEBT _t-1_	9.673E-10	.000	1.146	3.191	.004	.188	5.322
	$RESERVES _t-1_	-6.203E-9	.000	-1.029	-4.030	.001	.372	2.690
	PS/AV _t-1_	-6.759	7.609	-.297	-.888	.384	.217	4.600

Dependent Variable: CRISIS _t_. R^2^ = .467. F = 6.417 (.003).

The predictor variables $Debt and $Reserves show statistically significant relationships with the dependent variable CRISIS. Specifically, for every one unit increase in $Debt, CRISIS is expected to increase by approximately trivial 9.673E-10 units (p = 0.004). Similarly, for every one unit increase in $Reserves, CRISIS is expected to decrease by approximately minor -6.203E-9 units (p = 0.001).

However, the predictor variable PS&AVT does not exhibit a statistically significant *direct* relationship with CRISIS (p = 0.384). Therefore, the results suggest that changes in PS&AVT do not have a meaningful direct impact on the value of CRISIS (Table 3).

#### Political stability

Political Stability and absence of violence/Terrorism on CRISIS did not show any significant impact. The regression analysis reveals that PS&AVT accounts for approximately 55.5% of the variance in $Reserves (R Square = 0.555). The coefficient for PS&AVT indicates that a one-unit increase in PS&AVT would result in an increase of approximately 2815632325 $Reserves. This relationship is statistically significant (p < 0.000). However, the presence of positive autocorrelation in the residuals (Durbin-Watson = 0.578) suggests the need for further investigation (Table 4).

**Table 4:** PS&AV on $Reserves.

		Unstandardized Coefficients		Standardized Coefficients	t	Sig.	Collinearity Statistics	
		B	Std. Error	Beta			Tolerance	VIF
1	(Constant)	7051892867	591877314		11.914	.000		
	Political Stability and Absence of Violence96	2815632325	514972774	.745	5.468	.000	1.000	1.000

Dependent Variable: $RESERVES. R2 = .555. F = 29.89 (< .000).

PS&AVT on Debt (Table 5): The regression analysis reveals that PS&AV explains approximately 77.5% of the variance in $Debt (R Square = 0.775). The coefficient for PS&AVT of 23,767,977,200.367 indicates that a one-unit increase in PS&AV is associated with approximately $23,767,977,200.367 in $Debt. This relationship is statistically significant (p < 0.000). The ANOVA results confirm the overall significance of the regression model (p < 0.001).

**Table 5:** PS&AVT on $Debt.

		Unstandardized Coefficients		Standardized Coefficients	t	Sig.	Collinearity Statistics	
		B	Std. Error	Beta			Tolerance	VIF
1	(Constant)	48602343876	3005293635		16.172	.000		
	Political Stability and Absence of Violence96	23767977200	2614806080	.880	9.090	.000	1.000	1.000

Dependent Variable: $DEBT. R2 = .775. F = 82.624 (< .000).

### Simultaneity causality

Simultaneous causality issues occur when two or more variables in a model are interrelated and influence each other simultaneously, making it challenging to establish the direction of causality between them. Simultaneity bias can complicate efforts to estimate the true causal relationships between variables accurately. To address these issues of bidirectional causality and endogeneity, researchers often employ methods such as Granger causality testing, regression models incorporating lagged dependent variables and error terms (residuals), as well as scatter-plot charts. These tools help disentangle the complex relationships between variables and provide insights into the direction of causality.

Therefore, as additional support, the study incorporated a lagged CRISIS (t-1) variable to examine the reverse direction of the impact, revealing that the relationships were not statistically significant (see Table 6).

**Table 6:** Lagged DV regression- (CRISIS _t-1_ with the independent variables).

	Unstandardized Coefficients		Standardized Coefficients	t	Sig.	Collinearity Statistics	
	B	Std. Error	Beta			Tolerance	VIF
(Constant)	11.397	7.590		1.501	.147		
$DEBT _t_	4.592E-11	.000	.124	.317	**.754**	.211	4.748
$RESERVS _t_	-7.033E-10	.000	-.263	-1.042	**.309**	.504	1.984
PS & AV _t_	-4.499	3.933	-.438	-1.144	**.265**	.220	4.550

Dependent Variable: Lagged CRISIS _t-1_, R^2^ = .291. F = 3.015 **(>.05**).

#### Granger causality

The results of Granger causality Wald tests for potential $Debt Granger-Causes CRISIS show a statistically significant relationship (F(1, 20) = 16.416, p = 0.0006) suggesting that changes in $Debt Granger-cause changes in CRISIS, indicating that $Debt can serve as a predictive factor for variations in CRISIS. Testing Whether CRISIS Granger-Causes $Debt, the relationship was not found to be statistically significant (F(2, 20) = 0.40674, p = 0.6712). Thus, there is no strong evidence to support the notion that changes in CRISIS Granger-cause changes in $Debt. CRISIS does not appear to be a statistically significant predictor of variations in $Debt.

In relation to $Reserves, the results of the Granger causality Wald tests show a statistically significant relationship (F(2, 20) = 5.0459, p = 0.0168) suggesting that changes in the variable $Reserves Granger-cause changes in CRISIS. In other words, $Reserves can serve as a predictive factor for variations in CRISIS. Contrarily, test results for CRISIS Granger-Causes $Reserves was not found to be statistically significant (F(2, 20) = 1.5134, p = 0.2443), so no strong evidence to support the notion that changes in CRISIS Granger-cause changes in $Reserves.

The results of the Granger causality Wald tests for the causal relationships between the variable CRISIS and PS/AV show that the relationship is not statistically significant (F(2, 20) = 3.1128, p = 0.0665). Therefore, there is no strong evidence to support the idea that changes in PS/AV Granger-cause changes in CRISIS. In other words, PS/AV does not appear to be a statistically significant predictor of variations in CRISIS. Also, the relationship was not found to be statistically significant (F(2, 20) = 0.57654, p = 0.5709) for CRISIS Granger-Causes PS/AV suggesting that changes in CRISIS do not Granger-cause changes in PS/AV (Table 7).

**Table 7:** Granger causality test results.

Equation	Excluded	F	df	df_r	Prob > F
CRISIS	DEBT	16.416	1	20	**0.0006**
DEBT	CRISIS	0.40674	2	20	0.6712
CRISIS	RESERVES	5.0459	2	20	**0.0168**
RESERVES	CRISIS	1.5134	2	20	0.2443
CRISIS	PS/AV	3.1128	2	20	0.0665
PS/AV	CRISIS	0.57654	2	20	0.5709

#### Endogeneity

Endogeneity is primarily a concern related to the independent variables in a regression analysis. It arises when one or more independent variables are correlated with the error term in the regression model, violating the assumption of exogeneity. Endogeneity can arise as a result of measurement error, reverse casualty/simultaneity, omitted variable or unobserved variables, omitted selection, lagged dependent variables. These are the main reasons why X and e might be correlated or main causes of failure of exogeneity [73].

Therefore, another regression analysis was conducted, incorporating the lagged CRISIS variable (t-1), an additional independent variable, as a control Variable. The results demonstrate that this inclusion does not alter the significance of the impact of any of the independent variables (t-1), except for PS & AV (t-1), on CRISIS (t), thereby providing additional support for the absence of endogeneity issue related to the majority of the independent variables (see Table 8).

**Table 8:** CRISIS _t-1_ as a control variable.

		Unstandardized Coefficients		Standardized Coefficients	t	Sig.	Collinearity Statistics	
		B	Std. Error	Beta			Tolerance	VIF
1	(Constant)	11.120	14.656		.759	.456		
	**CRISIS LGD**_**t-1**_	**-.755**	**.543**	**-.333**	**-1.392**	**.179**	**.406**	**2.461**
	DEBT _t-1_	1.235E-9	.000	1.455	3.507	.002	.135	7.418
	RESERVES _t-1_	-7.157E-9	.000	-1.180	-4.338	.000	.313	3.191
	PS & AV _t-1_	-15.365	9.693	-.670	-1.585	.128	.130	7.705

Dependent Variable: CRISIS _t._ R^2^ = .513, F = 5.525 (< .01).

Further, according to the results of regression on the error term (Residuals) of the original regression the independent variables do not have a significant impact on the error term (RESIDUAL) in the original regression model (Table 3) and it does not explain much variance in the error term (R^2^ = 0) and none of the independent variables are statistically significant predictors of the error term (p>.05). This suggests that there may not be strong bidirectional causality or endogeneity between these variables in the specified model (Table 9).

**Table 9:** Regression on the error term (Residual).

		Unstandardized Coefficients		Standardized Coefficients	t	Sig.	Collinearity Statistics	
		B	Std. Error	Beta			Tolerance	VIF
1	(Constant)	-23.447	14.957		-1.568	.131		
	DEBT	7.459E-12	.000	.012	.024	**.981**	.188	5.322
	RESERVES	-4.417E-11	.000	-.010	-.029	**.977**	.372	2.690
	PS & AV	-.040	7.647	-.002	-.005	**.996**	.217	4.600

Dependent Variable: RESIDUAL, R^2^ = .000, F = 000 (p = 1)

**Fig 4:**
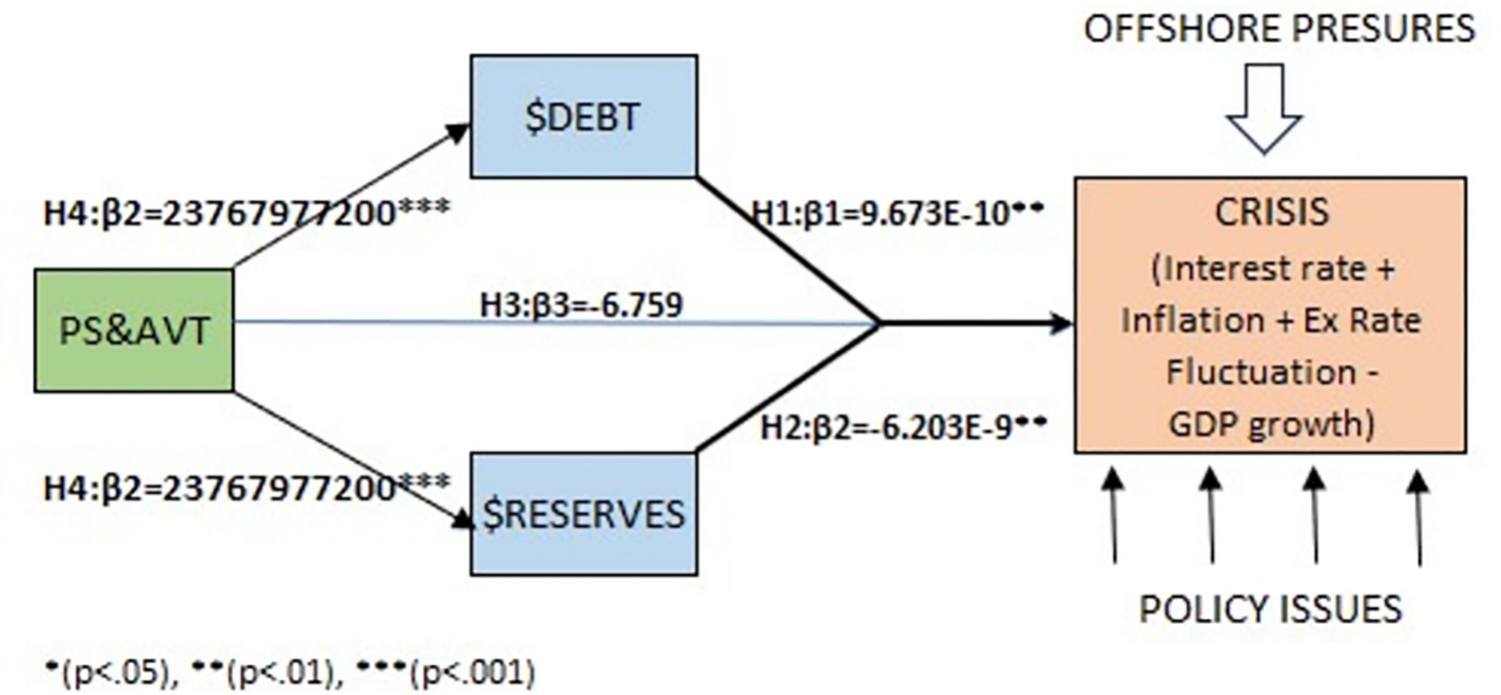
Independent variables Vs Regression residuals. •(p < .05), ••(p < .01), •••(p < .001).

The scatter plots depicting the relationships between the independent variables and the residuals provide additional evidence that supports the absence of endogeneity (Fig 4).

## Discussion

**Fig 5:**
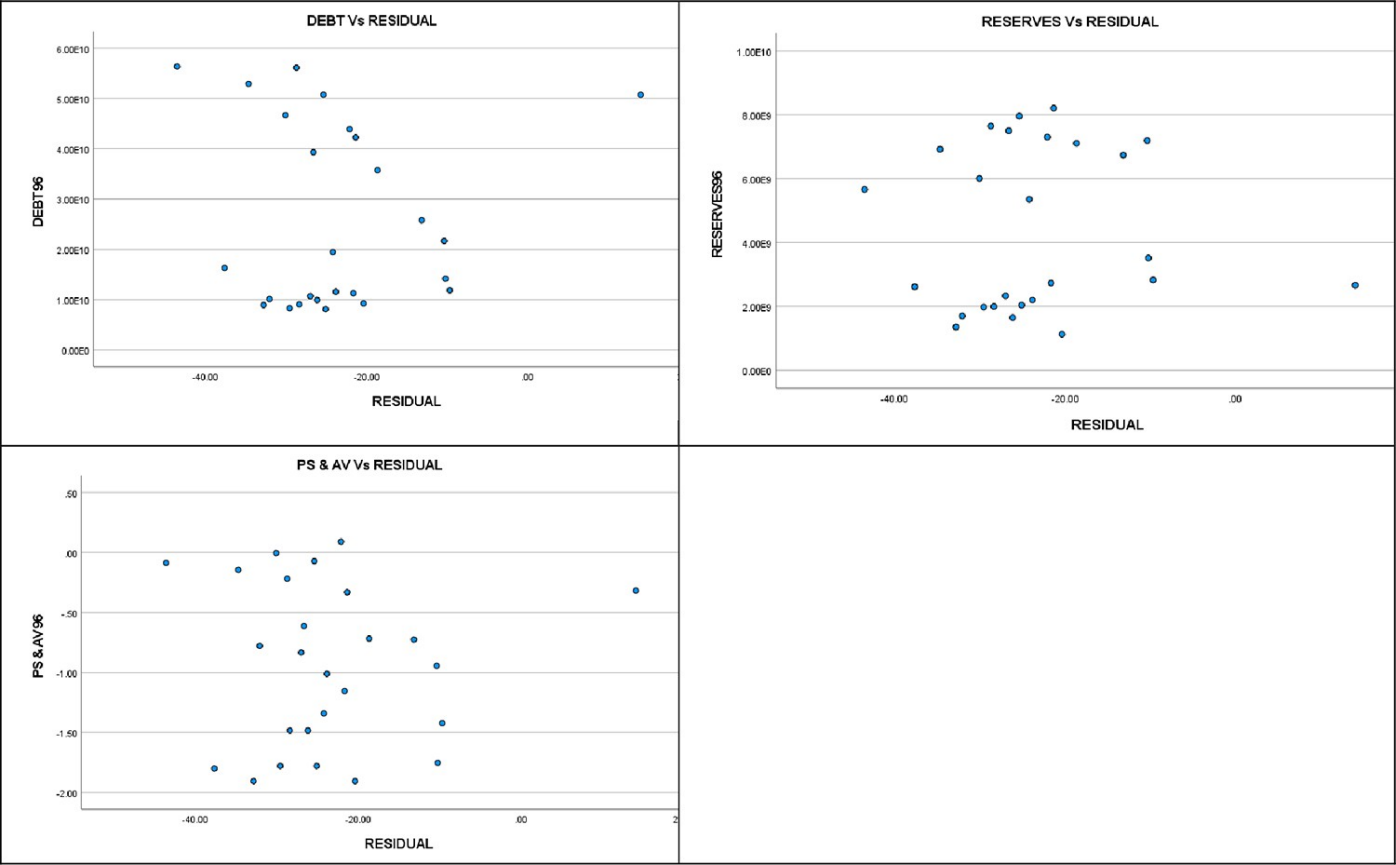
Regression results.

When $Debt and $Reserves are regressed together on CRISIS, they exhibit a moderately strong positive correlation although the magnitude is very small. The regression model is statistically significant (F =., p 0.00), highlighting the significant role of foreign reserves and external debt in explaining CRISIS. Increase in $Reserves is associated with a decrease in CRISIS and increase in $Debt is associated with increase in CRISIS. The PS&AVT shows positive strong association with $Reserves, means that PS&AVT indirectly influence negatively on CRISIS via foreign reserves. Similarly, the PS&AVT, as a predictor of $Debt, influences positively of $Debt, and therefore, indirectly influence negatively on CRISIS through external debt (Fig 5).

The Granger causality Wald tests revealed a significant causal relationship from $Reserves to CRISIS indicating that $Reserves can be used to predict changes in CRISIS. However, the reverse relationship, where CRISIS Granger-causes changes in $Reserves was not found to be statistically significant. The causal relationship from $Debt to CRISIS was significant, suggesting that $Debt can be used to predict changes in CRISIS. However, the reverse relationship, where CRISIS Granger-causes changes in $Debt was not found to be statistically significant. Also, there is no significant causal relationship between PV/AV and CRISIS. Changes in PS/AV are not found to predict changes in CRISIS. Likewise, CRISIS does not appear to significantly predict changes in PS/AV.

In terms of endogeneity, regression analysis on the error term (residuals) of the original regression model revealed that the independent variables in the original model did not exert a statistically significant impact on the error term (RESIDUAL). Furthermore, this analysis demonstrated that the independent variables did not account for a substantial proportion of variance in the error term, as evidenced by an R-squared (R2) value of 0. Importantly, none of the independent variables emerged as statistically significant predictors of the error term, with all p-values exceeding 0.05. The scatter plots depicting the relationships between the independent variables and the residuals provide additional evidence that supports the absence of endogeneity. These findings collectively indicate that the original regression model does not appear to suffer from endogeneity issues related to a significant correlation between the independent variables and the error term.

**Fig 6:**
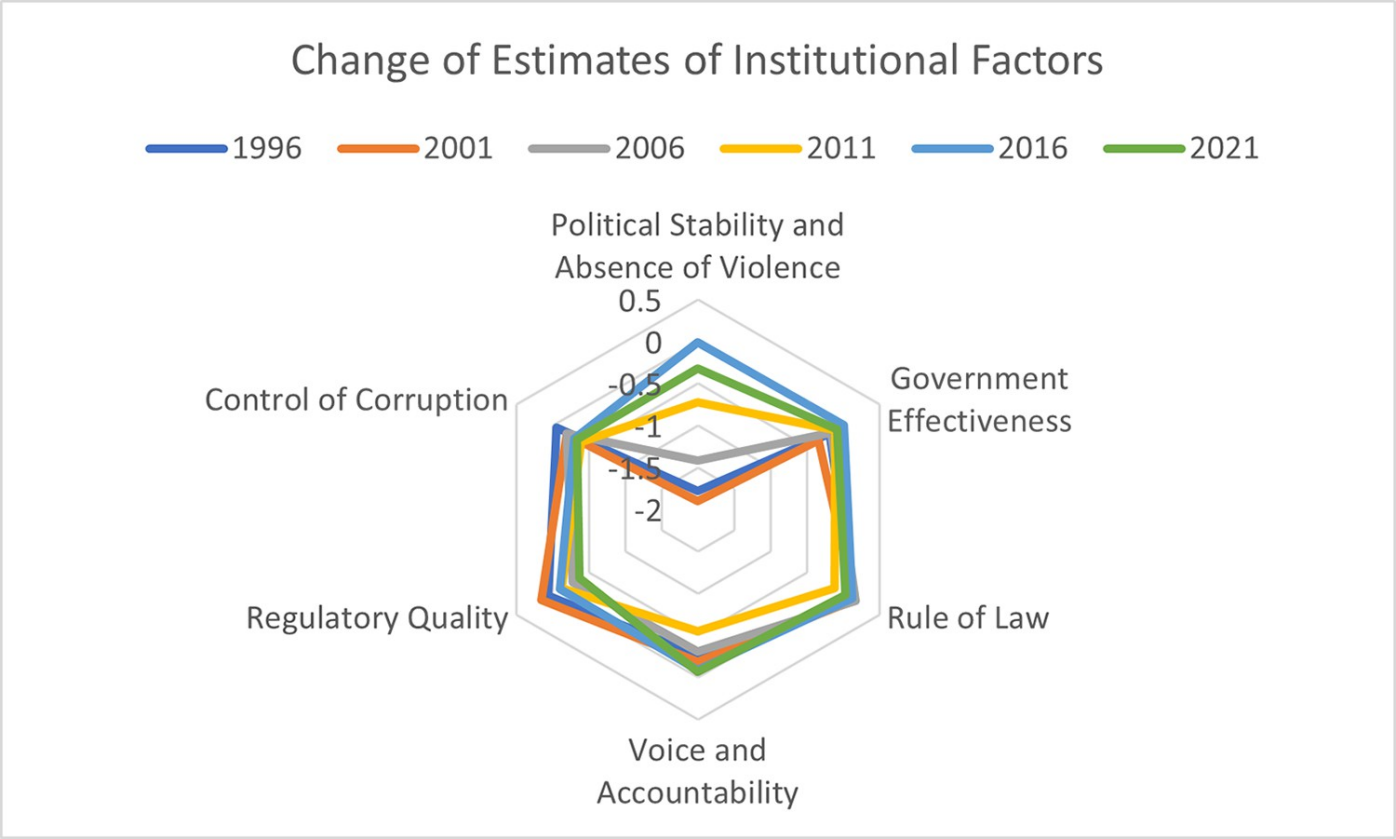
Change of estimates of WGI over 20 years. Source: Author’s illustration based on data from the World Bank.

When examining the change of the estimates of the six World Governing Indicators (WGI) over a span of 20 years, with data points taken at five-year intervals, PS&AVT shows the highest level of variation (Fig 6).

### A vicious cycle

**Fig 7:**
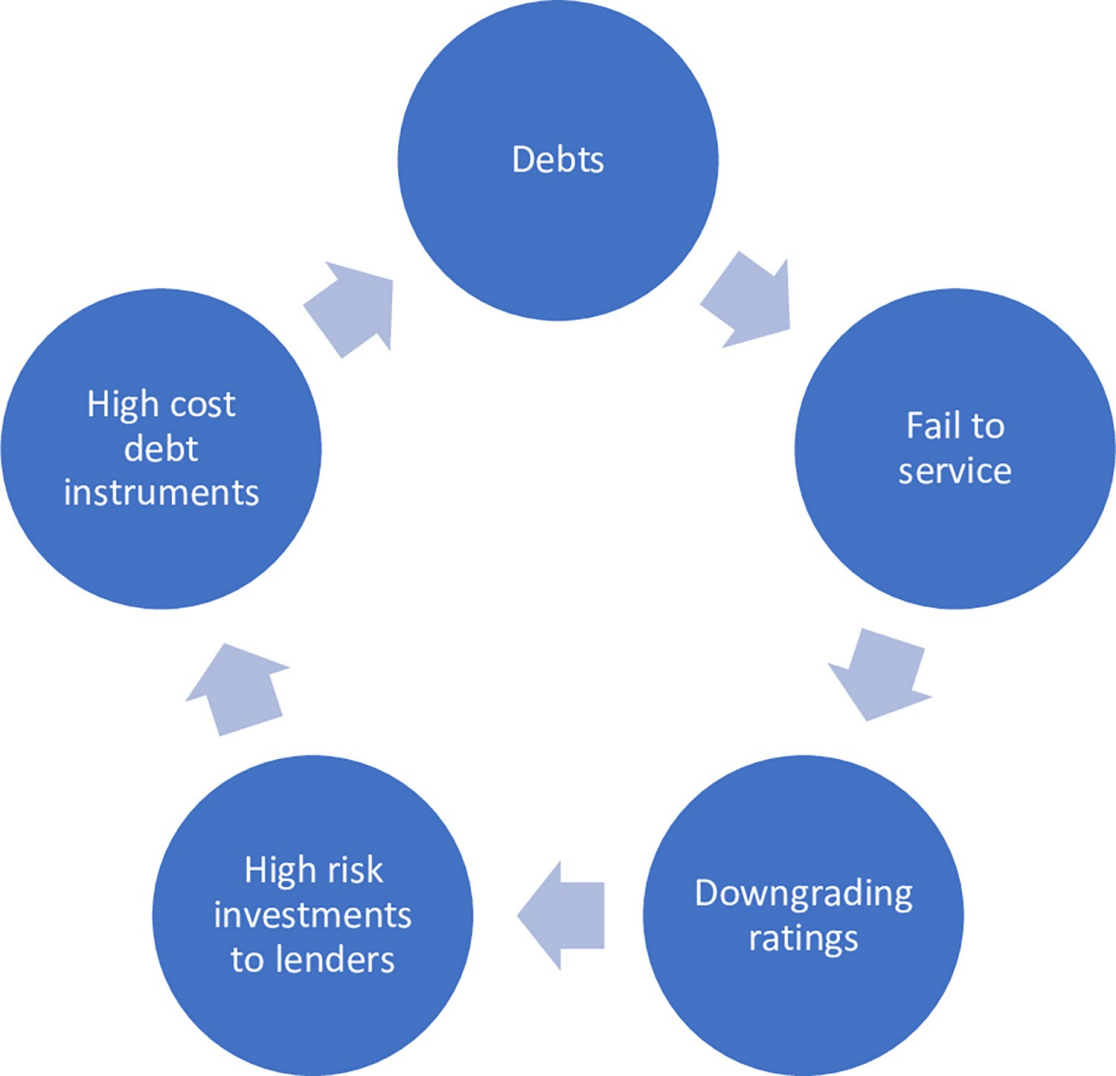
The vicious cycle. Source: Author’s illustration based on data from the World Bank.

The servicing of a large debt increases the expenditure of the government and leads to further deficits and borrowing. The cyclic relationship of the two leads to serious difficulties in containing either and results in economic instability and may even lead to a national financial crisis as happened in Sri Lanka in 2022 (Fig 7).

### Political stability

Starting in mid-January 2022, the availability of fuel experienced a significant decline, leading to the duration of daily blackouts of over 10 hours. Items that were previously considered essential, such as medicines and drugs, became scarce and elusive luxuries. Furthermore, the fuel crisis has posed a substantial threat to food supplies, primarily due to the shortage of diesel. This scarcity has hindered the operation of vehicles responsible for transporting essential commodities like vegetables, fruits, and fish.

In February, a significant number of Sri Lankans from all corners of the country took to the streets in unprecedented numbers, demanding the resignation of the president and his government. This mounting pressure culminated in a demonstration on 31 March, held directly in front of the president’s personal residence. In mid-April, Galleface belt in Colombo became the site of a large gathering, where protestors established a makeshift community known as Gotagogama, (Gota go village) amplifying the public’s demand for the president’s departure. After a three-month struggle, Gotabaya Rajapaksa became the first president in Sri Lanka’s to be ousted by a popular uprising. The protests can be seen as a reaction not only to the politics of nepotism but specifically to a type of nepotism that has resulted in the unraveling of the system [7]. He further predicts that “…so long as the Rajapaksas remain in power, on the frontlines or from the sidelines, instability will continue. That may debar Sri Lanka from tapping into much needed funds, which Colombo needs to get out of the crisis” [7, p. 5].

### Mismanagement

The financial crisis in Sri Lanka is an intricate and multifaceted issue, characterized by a convergence of various factors that have collectively contributed to its occurrence. At its core, the crisis can be attributed to a combination of weaknesses in the country’s financial systems, coupled with ill-suited fiscal and monetary policies. Additionally, sudden shifts and fluctuations in the global markets have further complicated the situation, exacerbating the existing vulnerabilities.

One pivotal aspect that cannot be overlooked is the mismanagement of borrowed funds, which has significantly contributed to the already burdensome debt load. Irresponsible handling of these funds has resulted in a snowballing effect, making the debt increasingly unsustainable and burdening the nation’s economy with escalating interest payments and repayment obligations. The misallocation of borrowed funds into non-productive ventures has hindered economic growth and stifled the potential for long-term financial stability.

The repercussions of the financial crisis extend beyond the realm of economics. They have permeated into various sectors of society, triggering widespread unrest, uprisings, and political instability. The frustrations arising from financial distress have fueled social discontent, leading to demonstrations, strikes, and protests, further complicating the country’s political landscape [74].

As the crisis deepened, it tested the resilience of institutions, governance, and public trust. The government’s ability to effectively manage the situation was put to the test, and in some instances, inadequate responses and delays worsened the crisis’s impact. Political instability, arising from both internal and external pressures, added another layer of complexity to the situation, hindering the implementation of comprehensive and sustainable solutions.

## Conclusions

The statistical analysis conducted in this study indicates a strong association between political stability, external debt and foreign reserves. These independent variables explain a high proportion of the variance in crisis which represented by a proxy consist of interest rate, inflation, currency devaluation adjusted to GDP growth. Granger causality tests showed predictive value for $Debt and $Reserve in relation to CRISIS, but the reverse relationship was not significant. Regression analysis using the error term and scatter plots supports the absence of endogeneity issues in the model.

This study advances our knowledge of the factors contributing to financial crises in Sri Lanka by revealing the intricate connections between external debt ($Debt), foreign reserves ($Reserves), political stability & the absence of violence/terrorism (PS & AV). It emphasizes that while the variable PS & AV may not have a direct impact on financial crises, its influence through $Debt and $Reserves should not be underestimated. The research contributes to the field by offering a more holistic and nuanced perspective on the dynamics of financial stability in the country.

The crisis in Sri Lanka is a complex issue with multiple factors contributing to its occurrence. It can be attributed to a combination of factors, including weak financial systems, inappropriate fiscal and monetary policies, and sudden changes in global markets. However, the mismanagement of externally borrowed funds (Debt) has played a significant role in exacerbating the already unsustainable debt burden. These factors have not only led to financial instability but have also contributed to wider unrest, uprisings, and political instability and violence (PS & AV). Therefore, it can be argued that the mismanagement of Debt, and political instability may have contributed significantly to the financial crisis in Sri Lanka. The study acknowledges certain limitations, such as the use of secondary data, potential omitted variable bias, and the focus solely on the case of Sri Lanka. These limitations should be considered when interpreting and generalizing the findings.

The research findings can be valuable for policymakers and politicians, providing insights into the significant impact of different independent variables on the crisis. The study emphasizes the importance of continued research into the complex factors contributing to financial crises and the consideration of different sets of independent variables when formulating policy.

## Supporting information

S1 Dataset (XLSX)

S1 File (PDF)
