# Supplementary material for: Impact of debt, reserves, and political stability on Sri Lanka’s financial crisis
Source: PLoS One. 2023 Nov 17;18(11):e0294455. doi: 10.1371/journal.pone.0294455 (PMC10656016; doi:10.1371/journal.pone.0294455)
Supplement: S1 File — (PDF) [file pone.0294455.s002.pdf]

S2 Supporting Information

I am very grateful that you pointed out these lapses. Please disregard my previous mail. I provide the missing information below. Also, in an Excel worksheet attached.

1.) **The values behind the means, standard deviations and other measures reported**.

The data set is provided in the file "S1 Dataset"

2.) **The values used to build graphs**.

Graph 1 (Fig 1: $Debt and $Reserves of Sri Lanka from 1997 to 2022 compared to the exchange rate)

Year	$Reserves	$Debt	Exchange Rate
1996	1,937.	8,486.0	0.0176
1997	2,029.0	8,197.0	0.0163
1998	1,984.0	8,749.0	0.0148
1999	1,639.0	9,088.0	0.0139
2000	1,049.0	9,031.0	0.0125
2001	1,338.0	8,372.0	0.0107
2002	1,700.0	9,333.0	0.0103
2003	2,329.0	10,735.0	0.0103
2004	2,195.8	11,346.0	0.0096
2005	2,735.0	11,353.8	0.0098
2006	2,836.7	11,981.4	0.0093
2007	3,508.2	13,989.5	0.0092
2008	2,560.9	15,106.6	0.0088
2009	5,357.4	18,662.1	0.0087
2010	7,196.5	21,437.9	0.0090
2011	6,749.3	32,747.9	0.0088
2012	7,105.8	37,098.1	0.0079
2013	7,495.0	39,905.3	0.0076
2014	8,208.4	42,914.1	0.0076
2015	7,303.6	44,839.4	0.0069
2016	6,019.0	46,418.0	0.0067
2017	7,958.7	51,603.9	0.0065
2018	6,919.2	52,411.8	0.0055
2019	7,642.4	54,811.5	0.0055
2020	5,664.3	49,041.3	0.0054
2021	3,139.2	51,775.1	0.0050
2022	1,897.6	49,678.1	0.0028

Graph 3 and 4 (Fig 2. CRISIS index fairly represent the situation in Sri Lanka)

YEAR	EX Volatility	12 TBILL	INFLATION	GDP GWT	CRISIS
1996	0.0712	10.01	9.45	5.1	14.4312
1997	0.0746	10.21	9.573696	6.4054	13.4529
1998	0.079043	12.59	9.364243	4.698423	17.33486
1999	0.105622	12.77	4.691706	4.30054	13.26679

2000	0.062841	18.22	6.176276	6.000033	18.45908
2001	0.223513	13.74	14.15846	-1.54541	29.66738
2002	0.044134	9.91	9.551032	3.964676	15.54049
2003	0.035848	7.24	6.314638	5.940269	7.650217
2004	0.004959	7.65	7.575926	5.445061	9.785823
2005	0.018195	10.37	11.63969	6.241748	15.78613
2006	0.030994	12.96	10.02018	7.668292	15.34289
2007	0.063259	19.96	15.84211	6.796826	29.06854
2008	-0.00682	12.12	22.5645	5.950088	28.72759
2009	0.05638	9.33	3.464963	3.538912	9.312431
2010	0.006408	7.55	6.217649	8.015967	5.75809
2011	-0.03314	9.31	6.716768	8.404733	7.588891
2012	0.027064	11.69	7.542914	9.144572	10.11541
2013	0.108476	8.29	6.90845	3.395733	11.91119
2014	0.035658	6.01	3.179002	4.960701	4.263959
2015	0.011094	7.3	3.768368	5.007683	6.071779
2016	0.088233	10.17	3.958888	4.486635	9.730487
2017	0.042695	8.9	7.704138	3.57817	13.06866
2018	0.026342	11.2	2.135038	3.272	10.08938
2019	0.15757	8.45	3.528394	2.32882	9.807144
2020	0.017962	5.05	6.153945	-3.61519	14.8371
2021	0.050455	8.24	7.014781	3.655632	11.6496
2022	0.524934	29.27	46.4	-7.8	83.99493

Graphs 3, 4 and 5 (Fig 5. Scatter-plots)

These charts are generated by SPSS software

Fig 6. Change of estimates of WGI over 20 years

Year	Political Stability and Absence of Violence96	Government Effectiveness96	Rule of Law96	Voice and Accountability96	Regulatory Quality96	Control of Corruption96
1996	-1.7783	-0.18363	0.16126	-0.26914	0.06473	-0.05654
2001	-1.90411	-0.34237	0.15953	-0.19072	0.158748	-0.19923
2006	-1.42155	-0.1598	0.177753	-0.31016	-0.27253	-0.1948
2011	-0.72477	-0.10306	-0.11706	-0.54505	-0.13135	-0.39936
2016	-0.00403	0.00942	0.125161	-0.08105	-0.10255	-0.30207
2021	-0.31793	-0.08303	0.038458	-0.06961	-0.36996	-0.33456

3.) The points extracted from images for analysis.

Fig 1. Fig 1: $Debt and $Reserves of Sri Lanka from 1997 to 2022 compared to the exchange rate.

This graph shows the comparative trends of external debt ($Debt) and foreign reserves ($Reserves) with exchange rate fluctuations (US$: LKR). It is referred to in the line 125 of the text in page 9.

Fig 2. CRISIS index fairly represent the situation in Sri Lanka

The point taken from this image is the appropriateness of the composite variable CRISIS to the current situation in Sri Lanka. This is referred in the line 277 of the text in page 15.

Fig 3. Theoretical framework

The wholistic approach is given in this image and referred to in line 299 of the text in page 16.

Fig 4. Independent variables Vs Regression residuals

The points extracted from these images are discussed in the line #428 in page 24, that is additional evidence to support absence of endogeneity.

Fig 5. Regression results

The regression results are discussed in lines from 431 to 439 in page 25.

Fig 6. Change of estimates of WGI over 20 years.

The points extracted from this image are discussed in lines 461, 462 and 463 in page 26.

Fig 7. The vicious cycle

The points extracted from this image are discussed in lines from 467 to 470 in page 26.
